# Eco-friendly bio-nanocomposites: pioneering sustainable biomedical advancements in engineering

**DOI:** 10.1186/s11671-024-04007-7

**Published:** 2024-05-09

**Authors:** J. Nandhini, E. Karthikeyan, S. Rajeshkumar

**Affiliations:** 1 Department of Pharmaceutics, Saveetha College of Pharmacy, Saveetha Institute of Medical and Technical Sciences, Chennai, 602105 India; 2 Department of Pharmaceutical Chemistry, Saveetha College of Pharmacy, Saveetha Institute of Medical and Technical Sciences, Chennai, 602105 Tamilnadu India; 3 Department of Pharmacology, Saveetha Dental College and Hospital, Saveetha Institute of Medical and Technical Sciences, Chennai, India

**Keywords:** Bio-nanocomposites, Biopolymers, Eco-friendly, Biomedical, Fabrication

## Abstract

Biomedical nanocomposites, which are an upcoming breed of mischievous materials, have ushered in a new dimension in the healthcare sector. Incorporating these materials tends to boost features this component already possesses and give might to things these components could not withstand alone. The biopolymer, which carries the nanoparticles, can simultaneously improve the composite's stiffness and biological characteristics, and vice versa. This increases the options of the composite and the number of times it can be used. The bio-nanocomposites and nanoparticles enable the ecocompatibility of the medicine in their biodegradability, and they, in this way, have ecological sustainability. The outcome is the improved properties of medicine and its associated positive impact on the environment. They have broad applications in antimicrobial agents, drug carriers, tissue regeneration, wound care, dentistry, bioimaging, and bone filler, among others. The dissertation on the elements of bio-nanocomposites emphasizes production techniques, their diverse applications in medicine, match-up issues, and future-boasting prospects in the bio-nanocomposites field. Through the utilization of such materials, scientists can develop more suitable for the environment and healthy biomedical solutions, and world healthcare in this way improves as well.

## Highlights

- Bio-nanocomposite composition explores the combination of natural biopolymers with nanoscale fillers.
- These materials have enhanced mechanical strength, thermal stability, and biocompatibility, making them ideal for tissue engineering, drug delivery, and diagnostic imaging.
- Their biodegradability and non-toxic nature minimize environmental impact, making them a game-changer in sustainable biomedical advancements.
- Acknowledges potential issues and the need for further research to ensure compatibility with biological systems.
- These materials can be easily tailored to specific requirements, enabling personalized medicine and targeted therapies.

## Introduction

The merging of nanotechnology and bio-composites is reshaping the context of innovation in the rapidly developing fields of materials science and healthcare [1]. The advent of bio-nanocomposites, a category of materials distinguished by their complex engineered structures, marks a new era in the quest for ground-breaking solutions in healthcare and beyond [2]. Bio-nanocomposites are advanced materials that combine biopolymers with nanoscale reinforcements, typically in the form of nanoparticles, nanotubes, or nanofibers in the 1–100 nm [3]. These composites harness the unique properties of both the biopolymer matrix and the nanoscale additives to create materials with improved mechanical, thermal, and barrier properties. The core of our investigation is an in-depth look at bio-nanocomposites [4]. These materials, known for their hybrid character, combine biopolymers, nanoparticles, and other components in a harmonic marriage. This analysis explores the various Bio-nanocomposites and their varying properties and applications. Preparation techniques, characteristics, functions, biocompatibility, biodegradability, and applications are only some of how bio-nanocomposites diverge from traditional nanocomposite and nanohybrid materials. Because of their nontoxicity, biocompatibility, complex engineered structures, and biodegradability, they have attracted much attention as a potential candidate for use in medical biomaterials [5]. Biomedical applications explored in this review include antimicrobial, anti-cancer, drug delivery, wound dressings, tissue engineering, anti-anemia, dental applications, bioimaging, and biosensors [6]***.*** The well-known biocompatibility and inherent nontoxicity of these compounds are the driving forces behind this preference. The filler and polymers must interact well to get the optimum results from bio-nanocomposites [7]. To create such a good relationship, it is essential to pick fabrication procedures appropriately based on the nature of the composite components. Significant manufacturing issues are commonly encountered when attempting to maintain long-term chemical compatibility between the polymer matrix and nanofillers while ensuring the even distribution of the nanofillers throughout the polymer matrix. [8]. Various conventional and cutting-edge methods can be used to fabricate bio-nanocomposites. Solution intercalation, melt interaction, in situ intercalative polymerisation, template synthesis, emulsification solvent diffusion, double emulsion solvent evaporation, electrospinning, ultrasonication, and using mammalian scaffold are some of the most commonly described techniques. These conventional procedures are often combined with others to mold the bio-nanocomposite into the required form [8].

Eco-friendly bio-nanocomposites are an innovative advancement in biomedicine, dealing with the increasing demand for sustainable healthcare solutions [9]. These biomaterials are developed by combining nanoscale fillers with biopolymers, showing high biodegradability, low adverse effects on nature, and a personalised drug delivery system. Their unmatched blend of biocompatibility and heightened performance underpins these compounds as vital elements for eco-friendly initiatives without sacrificing innovation [10, 11]. This review includes the basic principles of bio-nanocomposites, various preparation techniques, and their applications in biomedicine. It also discusses the challenges and future opportunities associated with bio-nanocomposites, including their biocompatibility and potential for future healthcare system change.

Highlights

- Bio-nanocomposite composition explores the combination of natural biopolymers with nanoscale fillers.
- These materials have enhanced mechanical strength, thermal stability, and biocompatibility, making them ideal for tissue engineering, drug delivery, and diagnostic imaging.
- Their biodegradability and non-toxic nature minimize environmental impact, making them a game-changer in sustainable biomedical advancements.
- Acknowledges potential issues and the need for further research to ensure compatibility with biological systems.
- These materials can be easily tailored to specific requirements, enabling personalized medicine and targeted therapies.

## Components of bio-nanocomposites

### Nanoparticles

Nanoparticles, which can be created from various materials, are commonly used to prepare biomedical bio-nanocomposites [12, 13]. Standard nanoparticles found in biological applications include: (i) Metallic nanoparticles like gold and silver, as well as magnetic metals, are commonly used in these disciplines [14]. Gold nanoparticles have many potential medical applications, including their use as contrast agents in medical imaging techniques like CT scans and their application in targeted medication delivery [15]. (ii) The biocompatibility of silica nanoparticles opens up various applications, such as drug delivery and imaging. Because of their porous composition, they can be infused with therapeutic or diagnostic substances [16]. (iii) Nanoparticles made of polymers: Biodegradable polymers (such as poly (lactic-co-glycolic acid), PLGA) are used to create nanoparticles that contain and release drugs in a controlled fashion [17]. These are commonly used to transport drugs to the body. Hyperthermia therapy, diagnostic imaging, and targeted medicine delivery all benefit from magnetic nanoparticles since these particles may be targeted to specific locations using an external magnetic field [18].

### Silica layers

Nanoclays, known as layered silicates, are stacks of small silicate platelets. The ability of these materials to reinforce polymers and impart unique features has led to their application in biomedical bio-nanocomposites [19]. Layered silicates improve the barrier properties of bio-nanocomposites, making them applicable in conditions like controlled-release drug administration and antibacterial wound dressings [20].

### Biopolymers

**Fig. 1:**
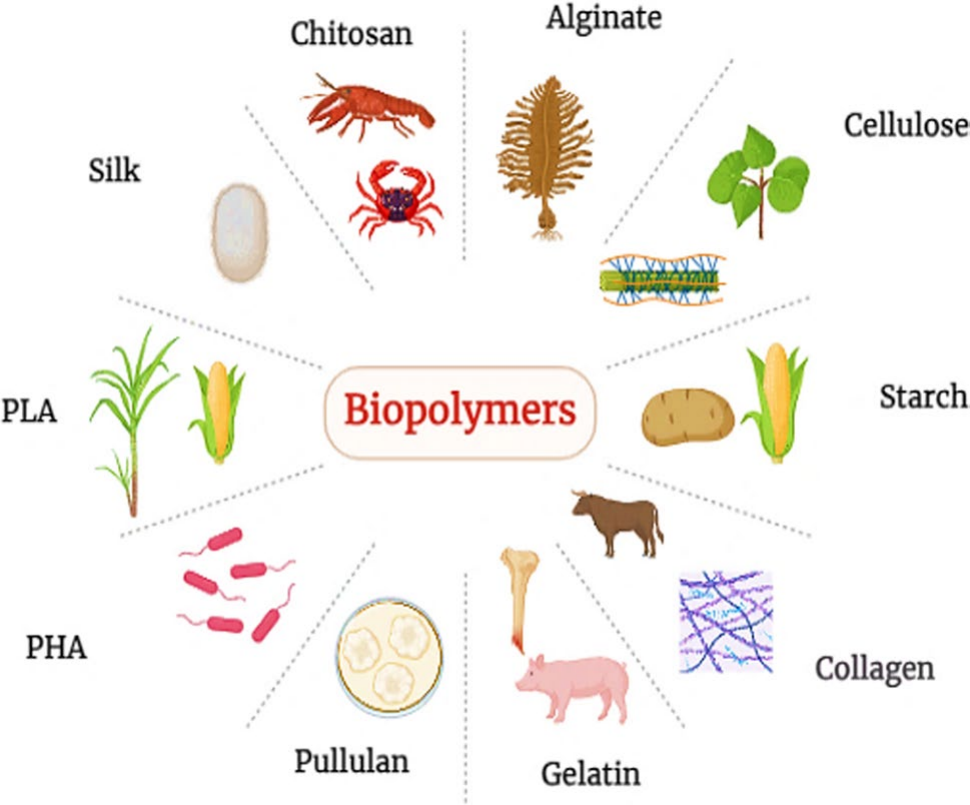
Types of bio-polymers

Biopolymers are naturally occurring, carbohydrate-based macromolecules selected for their biocompatibility, biodegradability, and adaptability. Their low toxicity and high stability make them ideal for a wide variety of applications in biomedical disciplines [21, 22]. Proteins and polysaccharides (glycans) are two of the most promising candidates for producing Bio-nanocomposites. Biopolymers are found in nature and include polysaccharides (such as chitosan, alginate, cellulose, starch, etc.), PHA (polyhydroxyalkanoates), and proteins [23]. Plants, algae, fungi, bacteria, and animals all contain polysaccharides, which have important structural and energy-storage roles in these organisms. They can be found in marine microbes and larger species. Synthetic polymers include poly-e-caprolactone (PCL), polyvinyl alcohol (PVA), polyglycolic acid (PGA), and polylactic acid (PLA) [24, 25]. Table 1 demonstrates different types of biopolymers, their origin, and biomedical applications. Figure 1 illustrates types of biopolymers.

**Table 1:** Types of biopolymers and their biomedical applications

S. no.	Biopolymer	Origin	Properties	Biomedical applications	Nanocomposite composition	Synthesis methods	Key properties	References
1	Chitosan	They are derived from chitin, a natural polymer found in the shells of crustaceans (shrimp and crab) and the cell walls of fungi	Biocompatible, biodegradable, antimicrobial, and has mucoadhesive properties	Drug delivery, wound healing, and tissue engineering	Chitosan-based nanocomposites	Electrospinning, Ionic Gelation	Enhanced mechanical strength, controlled drug release	[26]
2	Alginate	Extracted from brown seaweed and algae	Gel-forming, biocompatible, and capable of controlled drug release	Wound dressings, tissue engineering, and controlled drug delivery systems	Alginate-based nanocomposites	Ionic Gelation, Extrusion	Improved drug encapsulation, sustained release	[27]
3	Cellulose	Abundant in plant cell walls	Biocompatible, biodegradable, and possesses high tensile strength	Wound dressings, drug delivery, and scaffold materials for tissue engineering	Cellulose-based nanocomposites	Nanocrystal Blending, Solution Casting	Enhanced mechanical properties, controlled drug release	[28]
4	Starch	Derived from various plant sources, primarily corn, wheat, and potatoes	Biodegradable, readily available, and cost-effective	Drug encapsulation and controlled release	Starch-based nanocomposites	In-situ Polymerization, Melt Blending	Improved encapsulation efficiency, sustained release	[29]
5	Collagen	structural protein in the extracellular matrix of animals, typically sourced from bovine or porcine tissues	Biocompatible, biodegradable, and possesses excellent cell adhesion properties	Tissue engineering, wound healing, and cosmetic applications	Collagen-based nanocomposites	Self-Assembly, Freeze-Drying	Mimicked extracellular matrix, enhanced bioactivity	[30]
6	Gelatin	Derived from collagen through partial hydrolysis	Biocompatible, biodegradable, and provides a good matrix for cell growth	Drug encapsulation and tissue engineering	Gelatin-based nanocomposites	Crosslinking, Solvent Casting	Improved matrix stability, controlled drug release	[31]
7	Pullulan	It is produced by the fungus Aureo basidium pullulans	Biocompatible, water-soluble, and non-toxic	Drug delivery, wound healing, and as a coating material	Pullulan-based nanocomposites	Blending, Nanoprecipitation	Enhanced solubility, sustained drug release	[32]
8	PHA (Poly hydroxyl alkanoates)	Various bacteria naturally synthesise it as intracellular storage granules	Biodegradable, thermoplastic and biomedical applications	Sutures, tissue engineering, and controlled drug delivery	PHA-based nanocomposites	Melt Blending, Solvent Casting	Tunable mechanical properties, controlled drug release	[33]
9	PLA (Polylactic Acid)	Produced from renewable resources like corn starch or sugarcane	Biodegradable, biocompatible, and has good mechanical properties	Tissue engineering, drug delivery, and medical implants	PLA-based nanocomposites	Solution Blending, Melt Extrusion	Enhanced thermal stability, controlled drug release	[34]

#### Chitosan

Biopolymers like chitosan derived from chitin can be found in the cell walls of certain fungi and the shells of crustaceans like prawns and crabs. Chitosan's biocompatibility and biodegradability make it a versatile material with numerous potential applications [35]. Several amino (NH2) and hydroxyl (2OH) groups are present. It has a similar structure to cellulose and is also a linear copolymer. In addition, its significant amount of drug linkage capacity has made it an interesting candidate for drug delivery systems [36]. The application of mesoporous silica nanoparticles (MSNs) incorporated in chitosan and alginate biopolymer in bone tissue engineering is investigated in the work of Satar Yousefiasl et al. The scientists created and added MSNs to porous composite scaffolds based on alginate and chitosan biopolymer (Alg/Chit). The findings demonstrated that adding MSN enhanced swelling behaviours, reduced porosity, and increased mechanical strength. According to the study's findings, Alg/Chit/MSN bio-nanocomposites provide great promise for biological applications and the regeneration of bone tissue [16]. Wu et al. developed a method to prepare chitosan-modified magnetic nanoparticles (MNPs) for delivering doxorubicin (DOX). They created a composite using acetic acid using various techniques and analysed the MNPs/CS/DOX nanocomposites. The DOX was successfully loaded onto the MNPs/CS composite, and the DOX ratio increased as the CS and acetic acid concentrations increased. The MNPs/CS/DOX could sustain release over a longer period, indicating potential applications in magnetic drug carriers [37].

#### Alginate

It is well-known that alginate, a substance produced from seaweed, has gelling capabilities. It's a key ingredient in hydrogels that encapsulate cells for transformation, distributing drugs, and wound healing [38–40]. The use of alginate scaffold in articular cartilage repair was studied by Cheng et al. [41] Alginate hydrogels are investigated for their ability to promote chondrocyte and osteoblast proliferation and differentiation, facilitating cartilage healing and bone abnormalities. The implications of alginate-based hydrogels for regenerative medicine are discussed, along with their formulation and characterisation [16, 42]. The study performed by Amante et al. combines natural polymers with nanoemulsions (NEs) to create nanocomposites for wound dressings. Spray-drying produces alginate-pectin gelling powders as carriers for NEs loaded with curcumin, an antimicrobial drug. The encapsulation of NEs changes the size of microparticles, and the nanocomposites form a gel in less than 5 min. They promote wound transpiration, appear more elastic, and allow prolonged release of CCM-loaded NEs [43]. Andretti et al. developed nanocomposites to increase the retention time of nanoparticles in the intestinal tract using bio- and mucoadhesive matrixes. They produced tailored nanoemulsions (NEs) for encapsulating small hydrophobic drugs like tofacitinib. These NEs were efficiently internalised into human intestinal monolayers, reducing the inflammatory response. The resulting alginate hydrogel microbeads showed superior bioadhesive ability, promising potential for treating intestinal pathologies [44].

#### Cellulose

Cellulose is a natural biopolymer found in the cell walls of plants. It comprises linked glucose units and is Earth's most abundant organic compound [45]. It's biodegradable and can be used in sustainable materials, making it of interest for eco-friendly products and various biomedical applications [46]. To create bio-nanocomposites, cellulose nanocrystals are mixed with biodegradable polymers. Microfibrils and whiskers are the two simplest forms of nano-reinforcement in cellulose. Biomedical disciplines, such as bone tissue implantation, are paying much attention to cellulose-based bio-nanocomposites (bio-cellulose-based polyurethane nanocomposites) [47]. The study by Hesham Moustafa et al. prepared wound dressings from carboxymethyl cellulose/quaternized starch in the presence of antimicrobial cinnamon essential oil (CIEO) nanoemulsion. The bio-nanocomposites showed tensile properties, but hydrophilicity, water vapour, and oxygen barrier properties were affected. Cell viability was around 92–100%, and the nanoemulsions inhibited wound pathogenic microbes [48]. The study by Hong, Jung-ki et al. suggests that surface-oxidized cellulose nanocrystals (SO-CNCs) can improve the mechanical properties of poly(ε-caprolactone) (PCL) and induce biomineral formation upon PCL resorption. The researchers conducted an in vitro biomineralisation study and found that SO-CNCs were more effective at inducing mineral formation than SH-CNCs in simulated body fluid. Adding 10 wt% SO-CNCs increased Young's modulus and ultimate tensile strength by over twofold and 60%, respectively. According to these findings, SO-CNCs could serve as multi-functional nanoscale additives in PCL-based bone scaffolds [49].

#### Starch

Starch is a biopolymer that may be harvested from plants like maize and potatoes. It's a polysaccharide with many potential medical uses, from drug delivery to wound healing [50]. Nanocomposites derived from starch can create various drug delivery systems, including oral administration, injection depots, and transdermal patches [51]. They work well for medications that need controlled release over an extended period. By integrating nanoparticles into starch-based matrices, drug carriers can slowly and steadily release the drugs they carry, a process known as controlled drug delivery using starch-based nanocomposites [52]. Perlite/starch/single-walled carbon nanotube-glucose was ultrasonically synthesized and tested for delivery of drugs by Chegeni et al. [29]. At pHs 4.5 and 7.5, the curcumin-loaded bio-nanocomposite was evaluated against *E*. *coli*, *S*. *aureus*, and Candida albicans. Perlite/starch/single-walled carbon nanotube-glucose can transport drugs against bacteria and fungi under optimum conditions [29]. Prasad et al. synthesized copper oxide-rice starch nanocomposites from various copper and rice starch salts. The nanocomposites were characterized using FT-IR, XRD, SEM, TGA, and DSC spectroscopic methods. The nanocomposites formed a uniformly dispersed CuO nanoparticle network with smooth surfaces. They were thermally stable and exhibited significant antibacterial activity against various tested bacterial strains [53].

#### Collagen

Collagen is a natural biopolymer found in the extracellular matrix of connective tissues in animals, including humans. Biopolymers like collagen have found applications in drug delivery systems in film or discs, most notably in treating liver and corneal layer infections [54]. Composites of recombinant human morphogenetic protein matrix and collagen are excellent materials for constructing artificial bones [55]. Collagen-loaded β-tricalcium phosphate and calcium silicate were developed as a composite scaffold for tissue engineering applications to repair damaged bones [56]. The study uses bi-layered green electrospinning on polyvinyl chloride to explore the development of hybrid bio-nanocomposites containing collagen, Nigella sativa oil, and chitosan for tissue regeneration. Results show good fibres, moisture management, antibacterial activity, and wound healing [57]. The study by Villarruel et al. evaluated silica nanoparticles and silica-collagen nanocomposites for hGH loading and release. The results showed that silica nanoparticles had higher hGH adsorption capacity than thiol- and isobutyl-bearing particles. Silica-collagen nanocomposites showed a progressive protein release profile, reaching approximately 80% after 15 days. These findings suggest that biocompatible silica-collagen nanocomposites could be used for prolonged hGH delivery, potentially reducing the number of periodic administrations [58].

#### Gelatin

Gelatin is a natural protein that has the desirable properties of being both biocompatible and biodegradable. Since it is a cheap and easily accessible protein, it can be utilised in various applications [59]. Gelatin-based bio-nanocomposites are being used widely in medical and biological research. However, more cross-linkers are required to improve the physicochemical features of these nanocomposites [60, 61]. Gelatin hydrogel (mucoadhesive) nanocomposites are scaffolding for intravesical gene delivery. The increased penetration and preservation of lentiviral action made possible by these hydrogels allows for more in vivo transgenic expression of intravesical gene transfer [62]. The study investigated the stability of gelatin-curcumin nanocomposites on dental implants to prevent peri-implantitis. The nanocomposites were tested against dental pulp stem cells and showed a rapid release pattern for curcumin [63]. Konsek et al. developed a novel approach for producing a bioinspired dentine replacement material by cross-linking an apatite-gelatin nanocomposite material with various cross-linkers. The nanocomposites resemble mammalian dentine in composition and properties. Combining transglutaminase with casein improved the material's properties, resulting in a bioinspired material with similar properties to human dentine [64].

#### Pullulan

Pullulan is an extracellular natural polymer isolated from the polymorphic fungus Aureobasidium pullulans. Pullulan's structural flexibility and water-solubilizing characteristics are due to their exceptional linkage [65]. Recently, pullulan's role in manufacturing bio-nanocomposites has come into focus for its potential medical uses [66]. The report by Li et al. discusses the synthesis, characterisation, and bactericidal effectiveness of polymer-silver nanocomposite (PSN) films loaded with moxifloxacin (Mox). The films were prepared using solvent casting techniques and the synthesis of silver nanoparticles (AgNPs). The films showed good mechanical strength, elasticity, and swelling index. The highest bacterial susceptibility profiles were in PSN17, PSN18, and PSN20. The films showed quick recovery of infected dermal burn wounds in just seven days [67]. Yang et al.'s study demonstrates that tissue repair engineering using nanoparticles and stem cells can promote healing during tissue regeneration. Pul-Col-Au nanoparticles enhanced cell viability and anti-oxidative ability inhibited monocyte and platelet activation and induced the lowest cell apoptosis. The combination of pullulan, collagen, and Au nanoparticles could be potential nanocomposites for neuronal repair and skin tissue regeneration [68].

#### Polyhydroxyalkanoate (PHA)

Both gram-positive and gram-negative bacteria are capable of synthesising the linear biopolymer PHA. It looks like a synthetic polymer (thermoplastic). Because of its inherent biocompatibility and biodegradability, it finds application in various fields, including tissue engineering, wound healing, and drug delivery systems [69, 70]. Abdul et al. synthesised a biocompatible nanocomposite (PHA/Ch-WS2) with antimicrobial properties to improve therapeutics and reduce environmental impact. The nanocomposite was tested against *E*. *coli* K1 and MRSA using a time-kill method and cytotoxicity using HaCaT cell lines. The results showed significant bactericidal effects [71]. Mukheem et al. have developed a biocompatible nanocomposite based on polyhydroxyalkanoate, chitosan, and tungsten disulfide nanomaterial (PHA/Ch-WS2). The study aimed to investigate the antimicrobial activity of PHA/Ch-WS2 nanocomposites against multi-drug-resistant *E*. *coli* K1 and MRSA using the time-kill method. The cytotoxicity of the nanocomposite was evaluated using HaCaT cell lines using a lactate dehydrogenase assay. The results showed significant bactericidal effects, suggesting that the nanocomposite could be suitable for biomedical and sanitising applications without causing environmental harm [71].

#### Polylactic acid (PLA)

One of the most modern bioplastics is polylactic acid (PLA). Alternatively, you can call it polylactide. It's a linear thermoplastic polymer made from sustainable ingredients like corn or sugar beets [72]. Tissue engineering, the delivery of medications, and bone regeneration are a few of its biomedical applications [73, 74]. Nishat et al. developed an electrospun PLA-nHAP nanocomposite for drug delivery applications. The nanocomposite showed hydrogen bonds between nHAp and PLA, and its degradation was faster in PBS than in water. Cytotoxicity analysis showed a high survival rate of over 95% on Vero and BHK-21 cells. Gentamicin was loaded into the nanocomposite, and its in vitro drug release behaviour showed sustained release for 8 weeks, suggesting its potential as an antibacterial drug carrier in the dental and orthopaedic sectors [75]. The study by Lopresti et al. evaluated the physical and biological properties of polylactic acid (PLA) electrospun mats filled with nano silica and nano clay. Scanning calorimetry revealed the nucleating action of both nano silica and nano clay on PLA. Scaffolds were mechanically characterised, and cell culture assays were conducted to compare cell proliferation and morphology with neat PLA scaffolds. The results suggest that nano silica and nanoclay can be potential fillers for electrospun systems for bone tissue regeneration [76].

## Synthesis of bio-nanocomposites

**Fig. 2:**
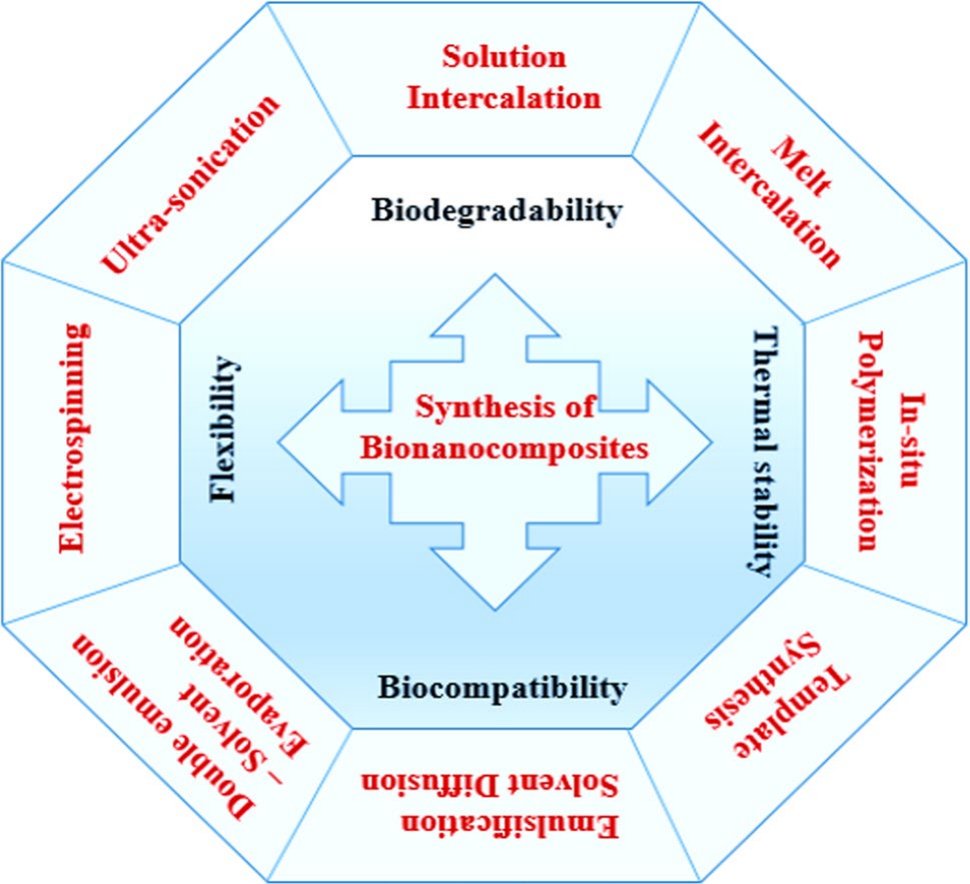
Different methods of fabrication of bio-nanocomposites

Including nanoparticles in a polymeric matrix is a simple way to make bio-nanocomposites. These bio-nanocomposites have better mechanical qualities, controlled drug-release capabilities, and increased biocompatibility [77]. Methods like solution casting and electrospinning often make these materials. Figure 2 illustrates different methods of fabrications of bio-nanocomposites.

These bio-nanocomposites have better mechanical qualities, higher biocompatibility, and controlled drug-release capabilities [78]. Because bio-nanocomposites can offer targeted therapy, enhanced structural support, and decreased toxicity, they are promising for use in biomedicine applications such as the delivery of drugs, tissue engineering, wound treatment, and medical implants [79, 80]. Using these bio-nanocomposites, antibacterial, analgesic, and anaesthetic properties might be achieved in transdermal drug administration [81]. Table 2 gives examples of different methods of fabrication of Bio-nanocomposites.

**Table 2:** Examples of different methods of fabrication of bio-nanocomposites

S. no.	Method of fabrication	Biopolymer	Filler material	Applications	References
1	Solution intercalation	Hydroxy-propyl methylcellulose (HPMC)	Montmorillonite (Cloisite®- Na +) clay mineral	Wound-dressing application	[82]
2	Melt intercalation	Poly (ϵ-caprolactone) (PCL)	Polylactic acid (PLA) nanoparticles loaded with triclosan	Trap triclosan and reduce burst release and long-term drug delivery	[83]
3	Template synthesis	Tannic acid—polyphenol	Silica matrix	Au@SiO2 and Ag@SiO2 nanocomposites possess high catalytic activity	[84]
4	Solvent casting	Chitosan and starch	Blend of chitosan and starch films	Controlled release of cefixime and wound healing	[51]
5	Double emulsion solvent evaporation	PGA-co-PDL (polyglycerol adipate-co-ϵ-caprolactone) and L-leucine	Model antigen BSA	Delivery of proteins to the lungs for immunostimulatory activity	[85]
6	Electro-spinning	Poly (lactic acid) (PLA)	Halloysite nanotubes	Promote rapid blood clotting and haemostasis when applied to wounds	[86]
7	Ultra-sonication	Polyimide	Zinc oxide nanoparticles	Antibacterial activity	[87]

### Solution intercalation

Proteins and starches are examples of biopolymers, and in this system, they are mixed with a soluble solvent. Solvents, including water, chloroform, and toluene cause inorganic microfiller silicate platelets to swell. When the biopolymer and swelling nanoparticle solution combine, the polymer chains intercalate and displace the solvent within the silicate interlayer. Removing the solvent while leaving the intercalated structure produces a layered silicate bio-nanocomposite [82].

### Melt intercalation

Melt intercalation has replaced previous manufacturing methods for polymer/layered silicate bio-nanocomposites. There are several advantages to both solution intercalation and in situ intercalative polymerisation. This process, which does not include using a solvent, combines the layered silicate within the polymer matrix through heating. Conventional procedures like extrusion and injection moulding are used to physically combine organophilic clay with a thermoplastic polymer at high temperatures. Nanocomposites are created by intercalating or exfoliating polymer strands. The method is widely employed for the production of thermoplastic nanocomposites. This technique can be helpful for polymers resistant to in-situ polymerisation and adsorption [83].

### In situ* intercalative polymerisation*

Between the intercalated sheets, polymer synthesis takes place. It is possible to use heat, radiation, diffusion of an appropriate initiator, or an organic initiator in situ to kick off the polymerisation process. This technique has been successfully extended to other thermoplastics after initially being used to make a nylon-montmorillonite nanocomposite. Thermoset-clay nanocomposites are a potential use for this method [8].

### Template synthesis

Forming silicates in an aqueous solution containing biopolymers is simple, easy, and flexible. These silicates are the primary constituents of hydroxide nanocomposites. However, this process rarely results in the formation of multilayer silicates. The precursor is a template to create water-soluble biomolecules, microbes, or whole-cell nanoparticles. Host crystals are nucleated and grow through self-assembled forces. The mesoporous matrix network finally captures the bioorganic nano template. The most prevalent methods for creating bio-nanocomposites, whether they contain inorganic or organic components, are polymer intercalation and sol–gel [84].

### Double emulsion solvent evaporation

The smaller dispersed phase is contained within the droplets of the dispersed phase in double emulsion(DE). The most common combinations are water–oil-water (w/o/w) DE and oil–water-oil (o/w/o) DE. Removing organic solvent from the dispersed phase by evaporation is the next step after forming the double emulsion (particulate dispersion). The active ingredient is encased in a rigid polymer because it causes a point of insolubility. Depending on the organic solvent's boiling point, the solvent can be evaporated under pressure using a rotary evaporator or stirred at room temperature. Xue Shen et al. developed a strategy to combine cancer diagnosis and treatment using nanocarriers like quantum dots, Fe3O4 nanocrystals, and doxorubicin. They created biodegradable poly (d,l-lactic-co-glycolic acid) polymeric nanocomposites using the double emulsion solvent evaporation method for tumour-specific targeting, drug/gene delivery, and cancer imaging. The nanocomposites showed remarkable synergistic antitumor effects in vitro and in vivo, with cell viability at around 14% and tumour volume decrease of 81% compared to saline. The study validates the potential of these multifunctional nanocomposites for cancer treatment [88].

### Electrospinning

Electrospinning is a flexible method for producing bio-nanocomposites, composite materials made of biodegradable polymers and nanofillers such as cellulose nanocrystals or clay nanoparticles [89]. A polymer solution containing the desired ingredients is electrostatically spun into nanofibers [90]. Bio-nanocomposites with improved structural and mechanical properties are produced when nanofibrous mats are formed by incorporating the nanofillers into the polymer matrix. The high surface area, precision control over fiber diameter, and biocompatibility of these materials make them useful in various contexts, such as tissue engineering, drug delivery, and wound dressing [18, 91]. Priyadarshini et al. investigated the effect of reinforcing nanofillers on drug release and the properties of nanofiller-drug-loaded nanofibrous systems. They isolated cellulose nanofibrils (CNF) from jute fibres and nanocollagen (NCG) from waste fish scales. The CNF-NCG bio-nanocomposites loaded nanofibrous structures showed excellent sustained release of ketorolac tromethamine for up to 16 h, making them excellent transdermal DDSs [92].

### Ultrasonication

Ultrasonication is a method for efficiently fabricating bio-nanocomposites for biomedical applications utilising high-frequency ultrasound waves. To evenly distribute nanofillers like nanoparticles across biodegradable polymers, ultrasonication can be used with high-frequency sound waves [93]. This method allows the size and location of nanofiller particles to be precisely controlled, leading to consistent dosing and distribution throughout the polymer matrix. The end product is bio-nanocomposites with enhanced mechanical and bioactive capabilities, which can be used in various biomedical settings, from drug delivery to tissue scaffolding and wound healing [94]. The study by Venkatesan et al. focuses on antimicrobial activity. They prepared SnO2 nanoparticles using chemical precipitation and reinforced them into poly(butylene adipate-co-terephthalate) (PBAT) using ultrasonication and solvent casting. The structural properties of the nanocomposites were analysed using various methods, showing an enhancement in mechanical strength over pure PBAT. The nanocomposites also demonstrated strong antibacterial activity against *E*. *coli* and *S*. *aureus* [95].

## Applications of bio-nanocomposites

**Fig. 3:**
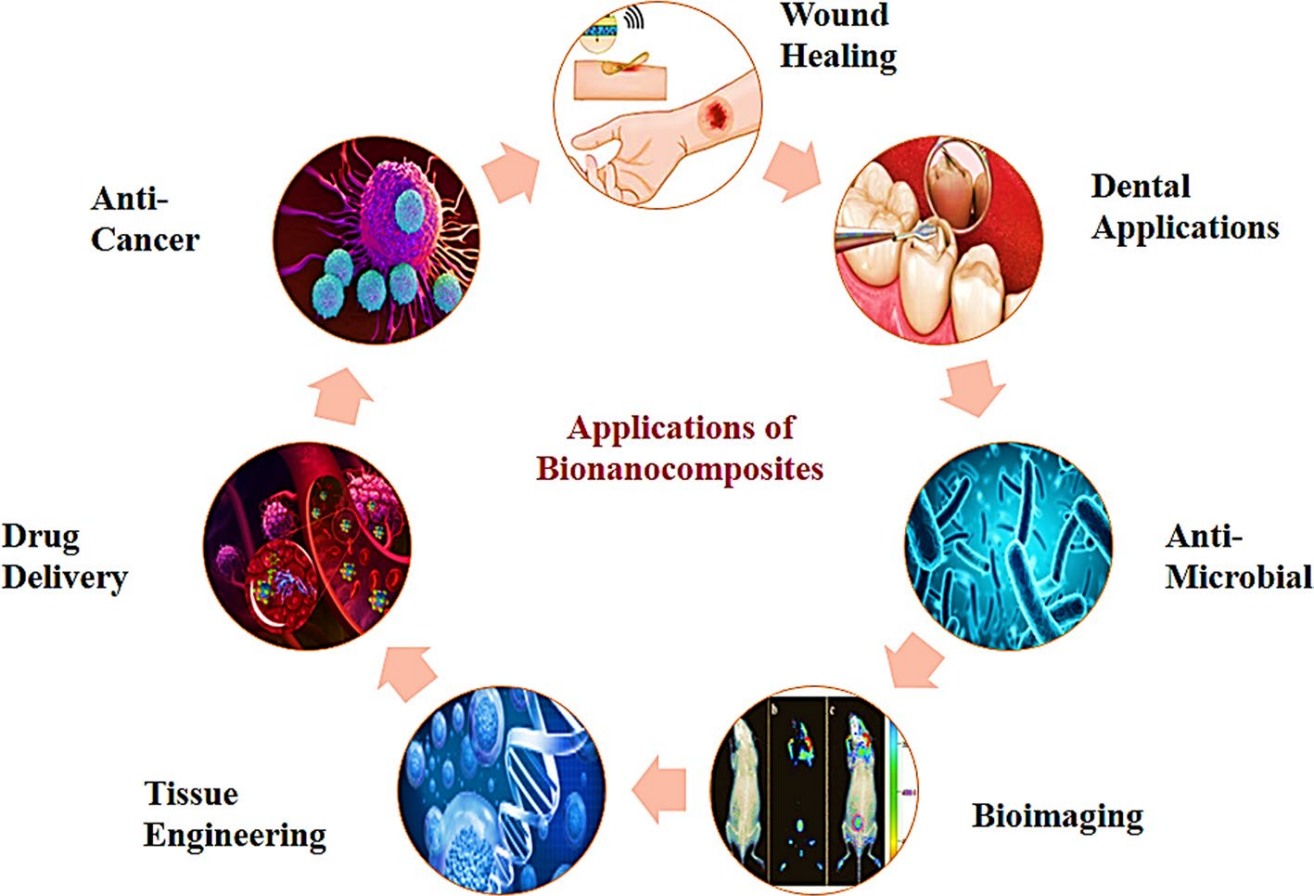
Applications of bio-nanocomposites

Bio-nanocomposites find diverse applications, including antimicrobial and anticancer functionalities, drug delivery systems, wound dressings, tissue engineering scaffolds, anti-anaemia treatments, dental applications, and bioimaging technologies, as illustrated in Fig. 3 and Table 3 shows diverse biomedical applications of Biopolymer-Based Nanocomposites.

**Table 3:** Diverse biomedical applications of biopolymer-based nanocomposites

S.no	Nanocomposites	Nanocomposite composition	Synthesis methods	Biomedical applications	Key properties	References
1	Chitosan-poloxamer nanocomposite	Chitosan, poloxamer	Solvent casting, blending, etc	Drug delivery, wound healing, tissue engineering	Enhanced drug release, biocompatibility	[96]
2	Chitosan-carbon nanotube	Chitosan, carbon nanotubes	Electrostatic assembly, covalent bonding	Antibacterial coatings, biosensors, drug delivery	Improved conductivity, antibacterial activity	[97]
3	Chitosan-nanohydroxyapatite nanocomposite	Chitosan, nanohydroxyapatite	Electrospinning, precipitation, etc	Bone tissue engineering, dental applications	Enhanced bone regeneration, bio-mineralization	[98]
4	Chitosan-silver nanocomposite	Chitosan, silver nanoparticles	Ionic gelation, co-precipitation, etc	Wound dressings, antimicrobial applications	Antimicrobial activity, sustained release	[99]
5	Chitosan-graphene oxide nanocomposite	Chitosan, graphene oxide	Electrostatic assembly, covalent bonding	Neural tissue engineering, drug delivery	Improved mechanical properties, enhanced drug release	[100]
6	Alginate- chitosan nanocomposite	Alginate, chitosan	Ionic gelation, coacervation, etc	Wound dressings, controlled drug release	Sustained drug release, enhanced stability	[101]
7	Alginate-silver nanocomposite	Alginate, silver nanoparticles	Ionotropic gelation, co-precipitation	Antimicrobial wound dressings, infection control	Antimicrobial activity, controlled release	[102]
8	Alginate-magnetic nanocomposite	Alginate, magnetic nanoparticles	Co-precipitation, emulsion methods	Magnetic resonance imaging, targeted drug delivery	Magnetic targeting, imaging capabilities	[103]
9	Alginate-zinc oxide nanocomposites	Alginate, zinc oxide nanoparticles	Co-precipitation, hydrothermal synthesis	UV-blocking wound dressings, antibacterial applications	UV-blocking properties, enhanced antibacterial activity	[104]
10	Alginate-gold nanorods	Alginate-silver nanoparticles, gold nanorods	Seed-mediated growth, surface functionalisation	Photothermal therapy, drug delivery	Photothermal effect, controlled drug release	[105]
11	Cellulose-nanocellulose nanocomposite	Cellulose, nanocellulose	Acid hydrolysis, electrospinning, etc	Drug delivery, wound healing, tissue engineering	Improved mechanical properties, biocompatibility	[106]
12	Cellulose-silver nanocomposite	Cellulose, silver nanoparticles	In-situ reduction, impregnation	Antimicrobial wound dressings, infection control	Enhanced antimicrobial properties, stability	[45]
13	Cellulose-magnetic nanocomposite	Cellulose, magnetic nanoparticles	Co-precipitation, emulsion methods	Magnetic resonance imaging, targeted drug delivery	Magnetic targeting, imaging capabilities	[107]
14	Cellulose-gold nanocomposites	Cellulose, gold nanoparticles	In-situ synthesis, chemical reduction	Photothermal therapy, biosensing	Photothermal effect, biosensing capabilities	[108]
15	Cellulose-chitosan-silver nanocomposites	Cellulose, chitosan, silver nanoparticles	Co-precipitation, blending	Antibacterial wound dressings, infection control	Synergistic antibacterial activity, controlled release	[109]
16	Starch-clay nanocomposites	Starch, clay nanocomposites	Melt blending, solution casting, etc	Controlled drug release, food packaging	Improved mechanical strength, sustained release	[52]
17	Starch-silver nanocomposite	Starch, silver nanoparticles	Microwave-assisted synthesis, etc	Antimicrobial applications, wound healing	Enhanced antibacterial activity, stability	[110]
18	Starch-mesoporous silica nanocomposites	Starch, mesoporous silica nanoparticles	Co-precipitation, sol–gel, etc	Controlled drug release, bioimaging	Controlled release, imaging capabilities	[111]
19	Starch-zinc oxide nanocomposite	Starch, zinc oxide nanoparticles	Hydrothermal synthesis, precipitation	UV-blocking wound dressings, antibacterial applications	UV-blocking properties, enhanced antibacterial activity	[112]
20	Starch-Gold Nanocomposite	Starch, gold nanoparticles	Citrate reduction, surface modification	Photothermal therapy, drug delivery	Photothermal effect, targeted drug delivery	[113]
21	Collagen-hydroxyapatite nanocomposite	Collagen, hydroxyapatite	Self-assembly, freeze-drying, etc	Bone tissue engineering, orthopaedic implants	Mimicked extracellular matrix, enhanced bioactivity	[114]
22	Collagen-silver nanocomposite	Collagen, silver nanoparticles	In-situ reduction, co-precipitation	Wound dressings, antibacterial coatings	Antimicrobial activity, controlled release	[115]
23	Collagen-magnetic nanocomposite	Collagen, magnetic nanoparticles	Co-precipitation, emulsion methods	Magnetic resonance imaging, targeted drug delivery	Magnetic targeting, imaging capabilities	[116]
24	Collagen-gold nanorods	Collagen, gold nanorods	Seed-mediated growth, surface functionalisation	Photothermal therapy, bioimaging	Photothermal effect, bioimaging capabilities	[117]
25	Collagen-chitosan-silver nanocomposites	Collagen, chitosan, silver nanoparticles	Co-precipitation, blending, etc	Antibacterial wound dressings, infection control	Synergistic antibacterial activity, controlled release	[118]
26	Gelatin-graphene oxide nanocomposite	Gelatin, graphene oxide	Solvent casting, crosslinking, etc	Drug delivery, tissue engineering, wound healing	Improved mechanical properties, drug release	[119]
27	Gelatin-magnetic nanocomposite	Gelatin, magnetic nanoparticles	Co-precipitation, emulsion methods	Hyperthermia cancer treatment, targeted drug delivery	Magnetic targeting, controlled drug release	[120]
28	Gelatin-silver nanowires	Gelatin, silver nanowires	Electrospinning, co-electrodeposition	Antibacterial coatings, wound healing	Enhanced antibacterial activity, wound healing	[121]
29	Gelatin- titanium dioxide nanocomposites	Gelatin, titanium dioxide nanoparticles	Co-precipitation, hydrothermal synthesis	UV-blocking wound dressings, drug delivery	UV-blocking properties, controlled release	[122]
30	Gelatin-gold nanocomposite	Gelatin, gold nanoparticles	Citrate reduction, surface modification	Photothermal therapy, drug delivery	Photothermal effect, targeted drug delivery	[123]
31	Pullulan-gold nanocomposite	Pullulan, gold nanoparticles	Co-precipitation, electrostatic assembly	Cancer therapy, drug delivery	Targeted drug delivery, enhanced biocompatibility	[124]
32	Pullulan-magnetic nanocomposite	Pullulan, magnetic nanoparticles	Co-precipitation, emulsion methods	Magnetic resonance imaging, targeted drug delivery	Magnetic targeting, imaging capabilities	[125]
33	Pullulan-carbon nanotubes	Pullulan, carbon nanotubes	Co-precipitation, thermal treatment	Drug delivery, biosensing	Controlled drug release, biosensing capabilities	[126]
34	Pullulan-zinc oxide nanocomposite	Pullulan, zinc oxide nanoparticles	Hydrothermal synthesis, precipitation	UV-blocking wound dressings, antibacterial applications	UV-blocking properties, enhanced antibacterial activity	[127]
35	Pullulan-silver nanoclusters	Pullulan, silver nanoclusters	Green synthesis, reduction methods	Antibacterial coatings, drug delivery	Enhanced antibacterial activity, sustained release	[128]
36	PHA-montmorillonite nanocomposites	PHA, montmorillonite nanocomposites	Melt blending, solvent casting, etc	Wound dressings, tissue engineering, controlled drug release	Enhanced mechanical properties, sustained release	[33]
37	PHA-silver nanocomposite	PHA, silver nanoparticles	In-situ reduction, impregnation	Antimicrobial applications, medical implants	Antimicrobial activity, controlled release	[129]
38	PHA-graphene oxide nanocomposites	PHA, graphene oxide	Solution blending, freeze-drying, etc	Drug delivery, tissue engineering, wound healing	Improved mechanical properties, drug release	[69]
39	PHA-gold nanorods	PHA, gold nanorods	Seed-mediated growth, surface functionalisation	Photothermal therapy, bioimaging	Photothermal effect, bioimaging capabilities	[130]
40	PHA-chitosan-silver nanocomposites	PHA, chitosan, silver nanoparticles	Co-precipitation, blending, etc	Antibacterial wound dressings, infection control	Synergistic antibacterial activity, controlled release	[131]
41	PLA-calcium phosphate nanocomposite	PLA, calcium phosphate	Solvent casting, electrospinning, etc	Bone tissue engineering, drug delivery	Enhanced bioactivity, controlled release	[132]
42	PLA-magnetic nanocomposites	PLA, magnetic nanoparticles	Co-precipitation, emulsion methods	Magnetic resonance imaging, targeted drug delivery	Magnetic targeting, imaging capabilities	[133]
43	PLA-silver nanofibers	PLA, silver nanofibers	Electrospinning, co-electrodeposition	Antibacterial coatings, wound healing	Enhanced antibacterial activity, wound healing	[134]
44	PLA-zinc oxide nanocomposite	PLA, zinc oxide nanoparticles	Hydrothermal synthesis, precipitation	UV-blocking wound dressings, antibacterial applications	UV-blocking properties, enhanced antibacterial activity	[74]
45	PLA-gold nanocomposite	PLA, gold nanoparticles	Citrate reduction, surface modification	Photothermal therapy, drug delivery	Citrate reduction, surface modification	[135]

### Anti-microbial

Bio-nanocomposites, blending the microscopic prowess of nanomaterials with the biological realm, naturally possess antimicrobial capabilities. This convergence of science and nature holds promise for diverse applications, offering innovative solutions to combat microbial challenges across domains [136]. Maraguan et al. developed cellulosic wound dressings incorporating allantoin-infused zinc layered hydroxide (allant-ZnLSH). These materials demonstrated antimicrobial activity against *S*. *aureus*, showcasing the potential for infection control. The films exhibited suitable mechanical properties and water vapour transmission rates for effective wound healing [137]. Using yeast glucans to create flexible beta-glucan/nanostructured zinc oxide films is an easy and environmentally friendly process that Paolo et al. have created. After being characterised using various techniques, the films were tested for their antibacterial activities against Staphylococcus epidermidis and *Escherichia* *coli*. The study highlights the promise of a new bio-nanocomposite for creating cutting-edge wound-healing devices [138]. Cuadra et al. used zinc acetate dehydrate, and silver acetate was used in chemical synthesis to produce ZnO/Ag nanocomposites for the research. They made nanocomposites with varying amounts of silver, and scanning electron microscopy revealed that silver significantly reduced the grain size of the zinc oxide nanoparticles. *S*. *aureus* and *E*. *coli* were used to test zinc oxide's antibacterial properties, and the results showed that the compound effectively kills both types of bacteria [139].

### Anti-cancer

**Fig. 4:**
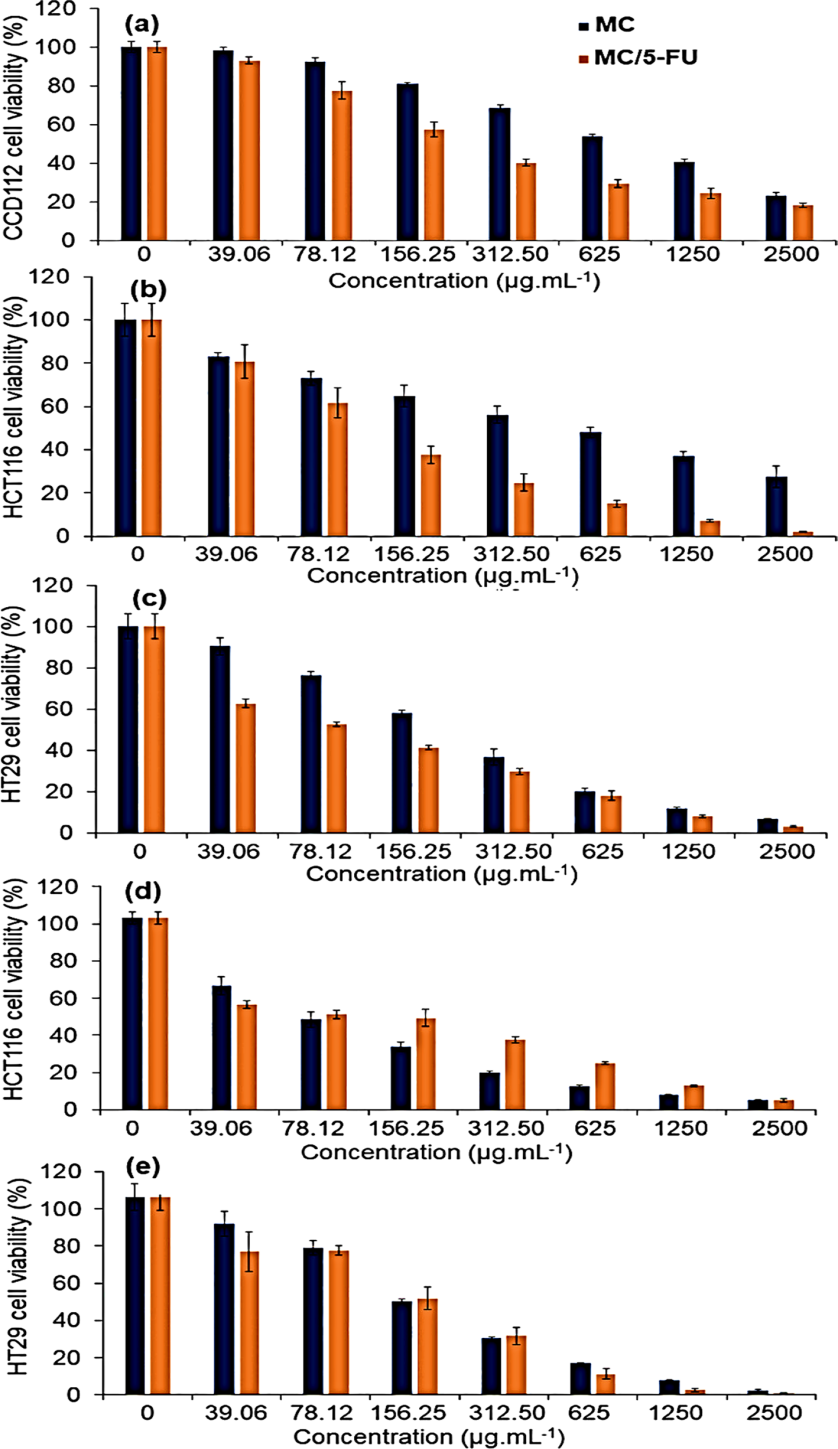
Anticancer activity of MC and MC/5-FU against **a** CCD112, **b** HCT116, and **c** HT29 2D monolayer models [144].

Traditional therapies for cancer, such as radiation treatment, surgery, and treatment with chemotherapy, are accompanied by unpleasant and often life-threatening side effects. The advantages of bio-based nanotechnology drug delivery systems are attracting the attention of researchers [140]. These systems have the potential to provide several benefits, including a large surface area, superior permeability, regulated drug release, and adequate encapsulation. Due to their amazing stability and enhanced circulation duration, nanomedicines can circumvent the negative effects of chemotherapy and other treatments. These nano-encapsulated bio-nanocomposites can potentially improve cancer treatment by facilitating more targeted activities against the disease [141, 142]. Polypyrrole was polymerized in situ with varying amounts of ZnO and grafted onto chitosan (Ppy/Z/C) to create a bio-nanocomposite matrix. The composite material's photocatalytic, antimicrobial, and cytotoxic characteristics improved significantly. In the presence of ultraviolet radiation, the composite proved to be an efficient catalyst in destroying reactive orange-16, CBB-R-250, and Methylene Blue. The Ppy/Z/C bio-nanocomposite shows promise in inhibiting the growth of human cancer cell lines (HeLa and MCF-7) and Gram-positive and Gram-negative bacterial pathogens. Evidence from apoptosis indicated striking efficacy against these cancer cell lines [143]. Annu et al. synthesised chitosan/polyvinyl alcohol (CS/PVA)–based zinc oxide and titanium dioxide hybrid bio-nanocomposites (BNCs) and compared their anticancer activity against skin cancer cell line A431. The BNCs showed higher free radical scavenging activity and higher inhibition zones against *Escherichia* *coli*. The study found 70% cancerous cell inhibition in ZnO BNCs compared to 61% in TiO2 BNCs [17]. Yusefi et al. developed MC/5-FU, a multifunctional magnetic polymer nanocomposite supported on rice straw cellulose, for colorectal cancer treatments. The nanocomposites showed enhanced drug release under various conditions and demonstrated anticancer effects against colorectal cancer cells. The easily synthesised MC/5-FU has the potential as a low-cost drug formulation. As shown in Fig. 4, the 2D monolayer of colorectal cancer cell lines HT29, HCT116, and CCD112 normal cells were inhibited by MC and MC/5-FU in a dose-dependent manner.

### Drug delivery

Bio-nanocomposites have been used in several drug delivery applications; these include clay, ceramic-based nanofillers, silicate nanoparticles, and carbon-based nanofillers, including graphene and carbon nanotubes (CNTs) [2]. The unique properties of CNTs, such as their high barrier crossing abilities, huge surface area, hollow core, and capacity to entrap multifunctional particles inside, make them an intriguing material. Clays have long been utilised as drug excipients due to their capacity to aid in the preservation of a stable suspension, emulsion, or drug adsorption [145]. Film-forming ability, bioadhesion, and cell absorption are just a few of the benefits that biopolymer nano clay composites provide. Beta-blockers and nonsteroidal anti-inflammatory medications have been regulated using microspheres made of clay bio-nanocomposites, including chitosan, alginate, and carboxymethyl cellulose (CMC) [146]. Table 4 Gives examples of some bio-nanocomposites used in drug delivery applications.

**Table 4:** Examples of some bio-nanocomposites used in drug delivery applications

Biopolymers/ natural materials	Nanoscale reinforcements	Drugs delivered	Key discoveries	References
Cellulose/Pectin	Nanoclays	5-methylindole derivative	Controlled release in the treatment of neurodegenerative diseases without exhibiting toxicity	[147]
Ethylcellulose/ Hydroxyl propyl methylcellulose	Halloysite nanotubes	Naproxen	Controlled delivery with reduced GI side effects	[148]
Crosslinked nano collagen-cellulose nanofibrils	Electrospun polyvinyl alcohol/ methylcellulose/polyethene glycol	Ketorolac tromethamine	Sustained release	[92]
Chitosan and starch	Chitosan and starch films	Cefixime	Antibacterial efficacy, with the highest drug permeation and highlighting the potential for controlled release	[51]
Poly(glycerol sebacic)-urethane (PGS-U)	Cloisite@30B	Metronidazole and tetracycline	Drugs exhibited sustained release over 200 h	[149]
I-carrageenan" and "gelatin	Montmorillonite inorganic clay mineral	Ciprofloxacin	Controlled drug release	[150]
Chitosan	Layered Double Hydroxide (LDH)	Tramadol HCL	Targeted and controlled drug release in the colon	[151]
Chitosan	Nanocellulose	Silver sulfadiazine and betamethasone	Advanced wound dressings with tailored properties for controlled drug release, antimicrobial activity	[152]
Chitosan and pectin	Halloysite nanotubes	Phenytoin sodium	Low release of phenytoin sodium in simulated gastric fluid and more controlled release in simulated intestinal fluid	[153]
Cellulose from rice straw	Ferrimagnetic iron oxide	5-Fluorouracil (5-FU)	Drug release is influenced by temperature and pH	[144]

Researchers have turned to magnetic polymer nanocomposites supported on rice straw cellulose to find new ways to treat colorectal cancer. Drug release was measured at different pH levels and temperatures, and it was discovered that Fe3O4-nanoparticles coat the cellulose matrix. Magnetic cellulose bio-nanocomposites loaded with 5-fluorouracil showed improved selectivity and anticancer effects against colorectal cancer cells, suggesting a possible, cost-effective clinical use in treating this disease [144].

In a study, bio-nanocomposite materials have been developed for controlled oral administration of a neuroprotective drug derivative of 5-methylindole, which is effective in treating neurodegenerative diseases like Alzheimer's. The drug was found to be intercalated in the clay's interlayer region, and its release was confirmed to be around 25% in acidic conditions. The materials were also tested as orodispersible foams [147]. Hydrogels made of oxidised starch and CuO have been designed to combat bacteria and respond to colon medication administration stimuli in a Namazi et al. study. It was determined that the hydrogels might be used for medical purposes, specifically to deliver naproxen to the colon. Hydrogel swelling behaviour was also studied, as it plays a significant role in drug release profiles. The swelling results in the produced samples correlated with the amount of added NPX [154].

### Wound dressings

Bio-nanocomposites exhibit potential as a viable platform for the regulated release of therapeutic chemicals and agents promoting healing. The rising prevalence of antibiotic resistance (AMR) has posed significant challenges in managing wound infections [136, 155]. Previous research has shown that a substantial proportion, namely 90%, of *S*. *aureus* strains obtained from wounds in healthcare settings around the globe exhibit resistance to penicillin [156]. *Staphylococcus* *aureus*, *Escherichia* *coli*, and Pseudomonas aeruginosa are often identified as the predominant bacteria in infected wounds, with a significant prevalence of resistance to one or several antimicrobial agents [157, 158]. This necessitates the use of sophisticated wound dressing remedies. Over time, wound dressings have transformed their functions and features. Initially, they were a passive barrier, protecting the injured site from the external environment. However, advancements in materials have enabled these dressings to participate in the healing process [159] actively. This active involvement is facilitated by the ability of these advanced materials to interact with the surrounding tissues and environment, both physically and biochemically. For example, they can initiate cellular proliferation pathways or eliminate bacterial infections [160]. Bio-nanocomposites (BNCs) have garnered significant interest due to their favourable biomedical characteristics. Bio-nanocomposites (BNCs) are a kind of composite materials in which the matrix is composed of biomacromolecules [161]. These biomacromolecules are generated from natural sources, possess bioactive properties, and can form films, hydrogels [162, 163], or fibers [164]. Indeed, conventional general wound dressings, such as sterile gauze, have been gradually substituted by novel dressings with improved healing properties and designed for particular wounds [165, 166]. For example, researchers have produced foams and hydrogels [167] specifically designed for wounds with high exudate levels [168]. On the other hand, hydrocolloids are often suggested for facilitating wound debridement. Films are prioritised where attributes such as flexibility, transparency, and gas permeability have significance, as seen in burn cases [169].

Esther Marugan and her team have developed allant-ZnLSH, a zinc-layered simple hydroxide and allantoin biohybrid, for potential wound dressing applications. The material was synthesised by adding zinc chloride to allantoin and characterised using various tests. The biopolymeric films showed suitable mechanical properties, water vapour transmission rates, and barrier properties against UV light. They also demonstrated antibacterial activity against *E*. *coli* and *S*. *aureus* [137].

**Fig. 5:**
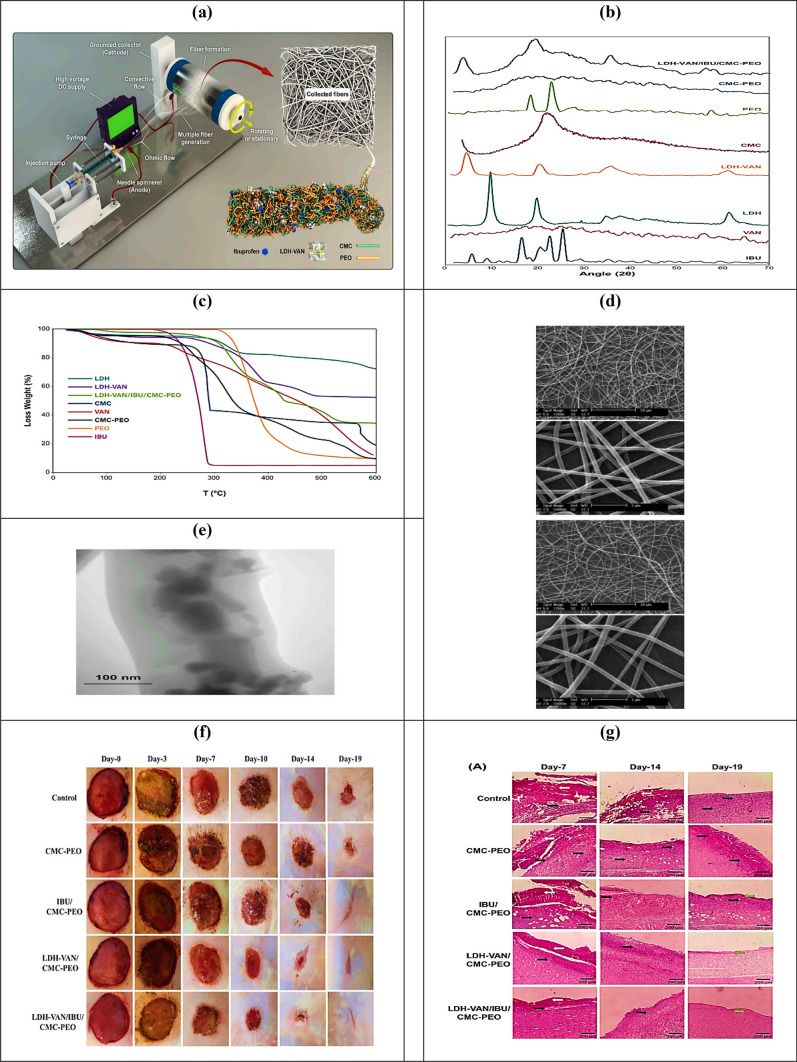
**a** Fabrication of nanofibers, **b** XRD, **c** Thermal behaviour, **d** SEM, **e** TEM, **f** Photographs of wounds treated by control, CMC-PEO, and IBU/CMC-PEO, LDH-VAN/CMC-PEO, and LDH-VAN/IBU/CMC-PEO groups on days 0, 3, 7, 10, 14, and 19 and **g** The histopathological photographs of wound tissue [91]

Sepideh et al. designed and developed ibuprofen (IBU) and layered double hydroxides-vancomycin (LDH-VAN) nanohybrid loaded bio-nano composite fibrous mats to increase wound healing rates. The nanohybrid particles were synthesised by in-situ incorporation of VAN into Mg-Al-LDH interlayers during hydroxide co-precipitation. The bio-nanocomposite fibrous mats were fabricated using the electrospinning technique and tested using various methods. Results showed no significant cytotoxicity on NIH/3 T3 fibroblast cells, and the wound area in rats treated with these mats was less than in other groups. Histological analysis showed that these mats exhibited faster wound healing than other nanofibrous mats [91]. Figure 5 illustrates (a) The electrospinning process of the preparation of nanofibers; (b) FTIR to show the interaction between test samples; (c) Thermal behavior of nanohybrid particles; d) SEM images of prepared CMC-PEO and LDH-VAN/IBU/CMC-PEO bio-nanocomposite fibrous mats of 224.32 ± 46.42 and 233.16 ± 49.08 nm, respectively; (e) TEM micrograph of LDH-VAN/IBU/CMC-PEO bio-nanocomposite fibrous mat containing 3 wt% of IBU and 6 wt% of LDH-VAN nanohybrid particles. (f) Photographs of wound healing activity in rats treated by control, CMC-PEO, IBU/CMC-PEO, LDH-VAN/CMC-PEO, and LDH-VAN/IBU/CMC-PEO groups on days 0, 3, 7, 10, 14, and 19 demonstrated a decrease in wound size after treatment, with the control and CMC-PEO groups showing a very slow healing process. (g) The histopathological photographs of wound tissue stained with Masson's trichrome of control, CMC-PEO, IBU/CMC-PEO, LDH-VAN/CMC-PEO, and LDH-VAN/IBU/CMC-PEO groups on the 7th, 14th, and 19th days. The black arrows show inflammatory cells, the white arrows indicate crusty scabs, and the green arrows point to re-epithelialization.

### Tissue engineering

Bio-nanocomposites are vital for tissue regeneration due to their amazing characteristics and outstanding mechanical strength, which result from the combination of hard and soft components [170]. They pave the way for novel approaches to matrix creation, regeneration of tissue, and drug delivery, all of which will hasten the development of advanced treatments [171, 172]. Good mechanical strength alongside good biocompatibility and bioactivity is a difficulty in creating bio-nanocomposites for tissue regeneration. Nanofillers like silicates, ceramics, inorganic nanoparticles, synthetic layered double hydroxides, carbon-based nanomaterials, and metal/metal oxides have been combined with biopolymers [172–175]. These nanofillers bring new capabilities, such as electrical conductivity and critical minerals for bodily processes. Because of their ability to integrate material, structural, and biological features on several levels, bio-nanocomposites are frequently utilised to simulate bone structure [176, 177]. Bone tissue engineering could potentially benefit from the use of synthetic polymers such as polylactic glycolic acid (PGA) [178], polylactic acid (PLA) [133], polycaprolactone [179], polyurethane [180], and polyvinyl alcohol (PVA) [181].

Researchers have discovered that porous composite scaffolds made from alginate and chitosan (Alg/Chit) can be modified by adding mesoporous silicon nanoparticles (MSNs) for bone/ tissue engineering. Mechanical strength and swelling characteristics favourable for cell adhesion, proliferation, and hydrolytic breakdown, were greatly enhanced by adding MSNs to the scaffolds. The Alg/Chit/MSN30 scaffolds were shown to be noncytotoxic and to improve cell viability considerably. Nanocomposite scaffolds containing MSN showed greater biomineralization characteristics than those of the Alg/Chit composite, indicating their potential for use in bone tissue engineering [16].

**Fig. 6:**
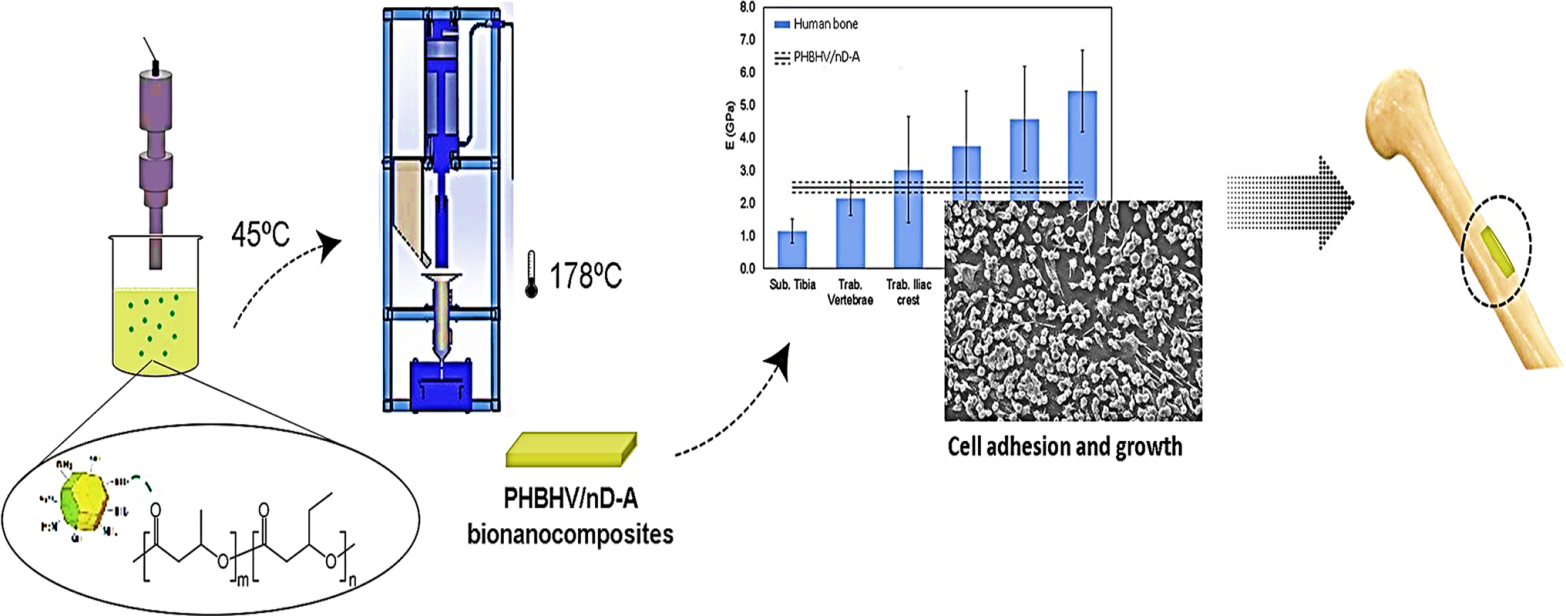
Injection-molded nanocomposites of poly(3-hydroxybutyrate-co-3-hydroxyvalerate) (PHBHV) with 6% 3-hydroxyvalerate (HV) and amino-nanodiamonds (nD-A in bone replacement applications) [182]

Maria et al. produced injection-moulded nanocomposites of poly(3-hydroxybutyrate-co-3-hydroxyvalerate) (PHBHV) with 6% 3-hydroxyvalerate (HV) and amino-nanodiamonds (nD-A) to investigate their mechanical and biological behaviour in bone replacement applications. The nanocomposites showed interactions between nD-A and PHBHV, with nD-A achieving satisfactory dispersion and distribution. Despite limited dispersion, PHBHV/2.0% nD-A had the best combination of E, strength, maximum deformation, highest glass transition temperature, and best adhesion coefficient and reinforcement effectiveness [182]. Figure 6 illustrates injection-molded nanocomposites of poly(3-hydroxybutyrate-co-3-hydroxyvalerate) (PHBHV) with 6% 3-hydroxyvalerate (HV) and amino-nanodiamonds (nD-A) in bone replacement applications.

### Anti-anaemia

Aoqiong et al. developed a stable and effective iron-WPI fibrils (Fe-FibWPI) nanocomposite for iron fortification. The nanocomposites showed improved antioxidant activity and higher in vitro iron bioavailability than native WPI fibrils. The Fe-FibWPI nanocomposites could be processed into liquid, gel, and powder forms, potentially enhancing their application in iron fortification in IDA management [183].

### Dental applications

Dental applications of bio-nanocomposites are intriguing because of their ability to replicate native tissue architectures while withstanding severe biting forces and abrasive conditions. Bio-nanocomposites combine biopolymers, including collagen, starch, alginate, polylactic acid, polyglycolic acid, and polycaprolactone, with inorganic materials for enhanced performance [184, 185]. The mechanical characteristics and biocompatibility of bio-nanocomposites make them suitable materials for dental tissue regeneration. Despite this, there is no agreement on whether or not nanomaterials are harmful, making nanosafety an important factor to consider. Mechanical characteristics and biological response can be enhanced using inorganic nanoparticles, allowing spatially regulated proteins to facilitate cellular adhesion and mineralized matrix nucleation [184, 186].

**Fig. 7:**
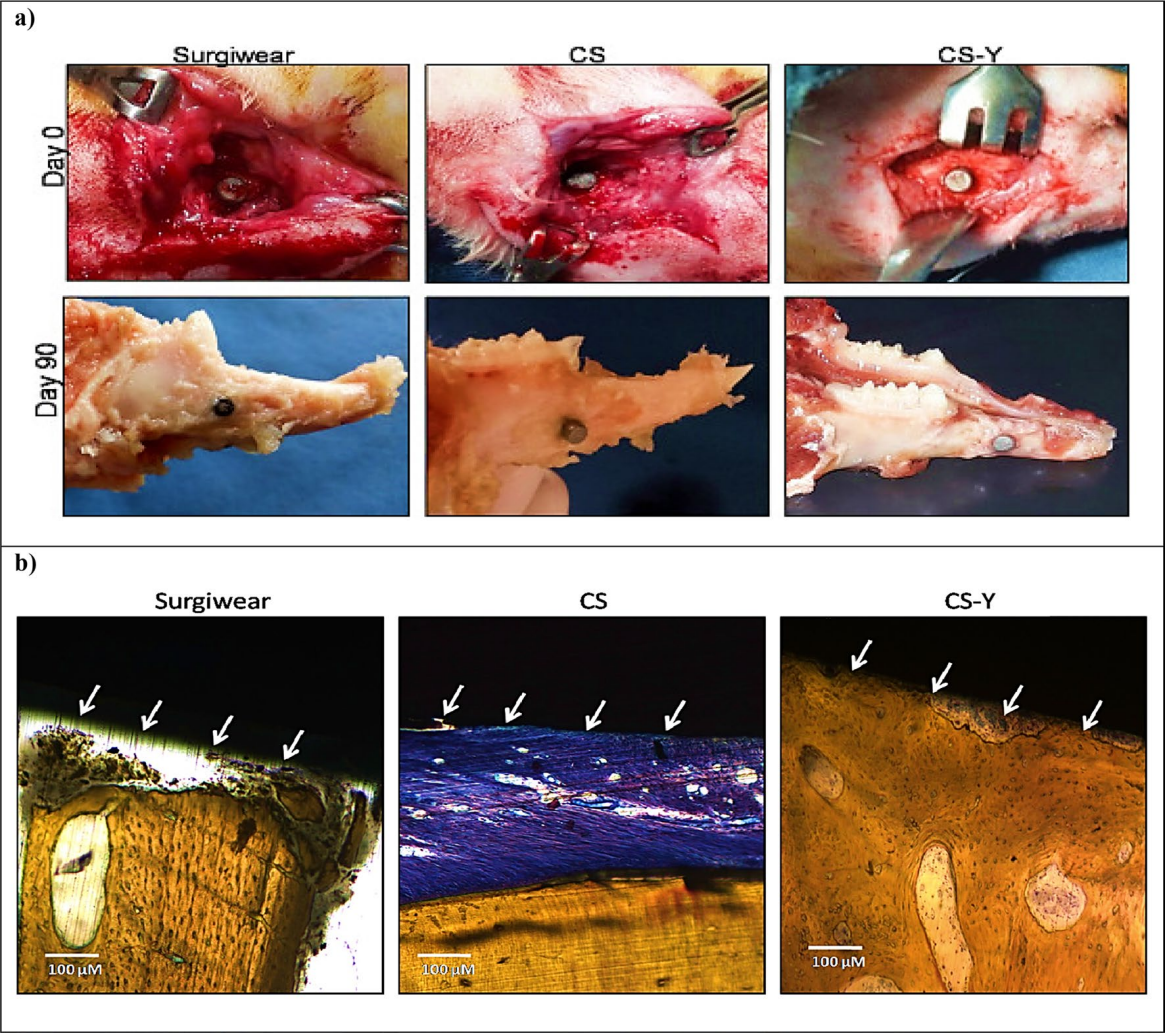
**a** Photographs of dental implants placed into the newly regenerated bone on day 0 (upper panel) and day 90 (lower panel) and **b** Histological analysis of newly formed bone around the dental implant on day 90 at low magnification (1 ×) (Stevenel's blue and van Gieson's picrofuchsin staining [187]

The study by Manju et al. developed a nanocomposite fibrous scaffold to promote osseointegration in rabbit mandibular defects. The scaffold was tested against autografts and a commercial nanoHA-collagen graft. The results showed that the scaffold was more effective in promoting new bone formation and osseointegration in rabbit mandibular defects than autografts and commercial grafts. This suggests that the nanocomposite fibrous scaffold is a promising biomaterial for edentulism patients [187]. Figure 7a illustrates photographs of dental implants placed into the newly regenerated bone on days 0 (upper panel) and 90 (lower panel) of the 3 groups. Surgiwear group (commercial graft without fibre), CS group (composite scaffold without fibre), and CS-Y group (composite scaffold with fiber) (b) illustrated histological analysis of newly formed bone around the dental implant on day 90 at low magnification (1 ×) (Stevenel's blue and van Gieson's picrofuchsin staining). CS-Y groups exhibited more compact and continuous cortical bone around the implant without fibrous tissue deposition.

Electrospinning and 3D printing have synthesised bio-nanocomposites containing biopolymers like silica, hydroxyapatite, halloysite, and gelatin for regenerating the dentin-pulp complex. Brown seaweeds are the source of alginate, a natural polysaccharide that is hydrophilic, biocompatible, and biodegradable. The periodontal ligament fibroblast cells' ability to connect to and multiply on gelatin bio-nanocomposites used as scaffolds for tissue engineering is demonstrated [186, 188].

Solmaz et al. investigated the stability of gelatin-curcumin nanocomposites on dental implants to prevent peri-implantitis. The nanocomposites were tested against dental pulp stem cells and showed a rapid release pattern for curcumin. The consistency of the coating was evaluated at intervals of one, 30, and 60 days. The nanocomposite was non-cytotoxic and maintained consistency for at least one month [63].

### Bioimaging and biosensors

Bio-nanocomposites have numerous uses in bioimaging and biosensors, providing novel strategies for better diagnosis and analysis. They are used as contrast agents in medical imaging procedures, including magnetic resonance imaging (MRI) [189] and computed tomography (CT) [190], to enhance diagnostic accuracy [191]. The signals emitted by these composites can also be used as fluorescent nanoprobes in fluorescence-based bioimaging, allowing for precise targeting of cells or biomolecules. Bio-nanocomposites serve a crucial role as coatings in biosensors, increasing the biocompatibility and stability of sensor surfaces [192]. They improve biomolecule binding, boosting the biosensors' sensitivity and specificity [193]. Biological compounds and pathogens can be detected with exquisite sensitivity thanks to incorporating quantum dots into bio-nanocomposites. Because of their conductive nanoparticles are also useful in electrochemical biosensors, which provide rapid and sensitive analyte detection without a label [194]. Bio-nanocomposites are at the cutting edge of developing bioimaging and biosensor technologies, with applications from point-of-care diagnostics to environmental monitoring and biological research [191, 193, 195].

## Biocompatibility of bio-nanocomposites

The investigation of biocompatibility pertains to examining how bio-nanocomposites interact with human body cells and tissues. Blood cell aggregation, hemolysis, and coagulation behaviours are examples of recent biocompatibility research employed with bio-nanocomposites [196].

### In vitro hemocompatibility

Bio-nanocomposites are subjected to in vitro hemocompatibility studies to see if they cause any release of haemoglobin into the plasma when they come into contact with red blood cells [197]. Materials designed to be biocompatible with the human body should not promote blood clotting, embolism, antigenic response, or the destruction of blood components. Because of this, blood biocompatibility testing is the gold standard for evaluating biomaterials. The thrombogenicity test is typically used to assess biomaterials wsith the hemolysis test. This test is a simple, sensitive, and widely used method for evaluating biomaterials' suitability for use with whole blood [198]. Nanjunda et al. synthesised the hydrogel composites for drug delivery and displayed cytocompatibility with HaCaT skin cells. In contrast, MCF-7 breast cancer cell viability decreased, hinting at potential anticancer effects. The study employed MTT assays to evaluate biocompatibility, revealing promising applications in medical contexts. C-30B, AgNPs, and lignosulfonic acid influenced the observed effects on cell viability [199].

### In vivo histocompatibility

Testing for cytotoxicity and biocompatibility in vivo includes determining the median lethal dose (LD50), doing haematological and serum chemistry analyses, analysing the pharmacokinetics of blood samples, and performing histopathological examinations. In vivo, tests for carcinogenicity are performed on mouse or rat models by OECD guidelines for testing chemicals [200], with the lethal dose of the test substance determined using probit analysis. Dose–response trials can be analysed using this technique by converting the percentage of animal deaths to probits. In 2017, Mahmood et al. created a novel cockle shell CaCO3 aragonite nanocomposite 3D scaffold implant. The quality of the regrown tissues was one major finding from the histological analysis. Histological analysis further supported the enhanced integration of the implanted materials to the host bone with substantial bone formation into and above the implanted scaffold without an excessive inflammatory response from the tissues [201].

## Challenges and future prospectives

There is great potential for innovation in using bio-nanocomposites in the medical field. Still, there are also several hurdles and exciting future opportunities associated with this field. However, it is still difficult to find nanofillers that are both effective and safe for use in the body [2]. Careful assessment of a nanoparticle's potential toxicity is required to guarantee it will not harm a patient. Getting innovative medical materials through regulatory approval can be lengthy and difficult. Establishing guidelines that can adapt to the ever-changing character of bio-nanocomposites is crucial. The reproducibility of bio-nanocomposites is vital for their widespread use. It's challenging to keep qualities constant throughout mass production [12]. The structural integrity and performance of bio-nanocomposites can be compromised if the nanoparticles utilised in the material move or agglomerate within the material [11]. Long-term dependability relies on getting a handle on these stability challenges. Due to the high price of nanoparticles and specialised manufacturing procedures, bio-nanocomposites might be difficult to mass produce. It's always difficult to find economical solutions that don't compromise quality [202, 203].

Future Bio-nanocomposites show promise as a tool for targeted drug delivery because their release of the medication may be precisely controlled, hence reducing adverse effects and maximising therapeutic gains. Artificial organs and tissue substitutes for regenerative medicine can be made from these composites because they can be designed to have the same mechanical and biological qualities as natural tissues [204]. Biodegradable bio-nanocomposites are being developed as a remedy to infections and inflammation caused by non-degradable implants, hence reducing the need for surgical removal [205]. Bio-nanocomposites, including bone implants and brain electrodes, can greatly improve their mechanical, electrical, and thermal properties by adding nanoparticles [179, 180]. Personalised medical devices and treatments that take into account individual differences are made possible by tailoring the properties of bio-nanocomposites for particular patients. It can improve healthcare diagnostics by creating ultra-sensitive tools and sensors for detecting and monitoring diseases at their earliest stages [206, 207]. Incorporating antimicrobial nanoparticles into bio-nanocomposites can potentially lessen the danger of infections caused by medical equipment. Improved diagnosis and treatment planning could result from enhanced tissue and organ visualisation using nanoparticles in bio-nanocomposites [193]. With increasing environmental concerns, it is important to produce biodegradable and eco-friendly bio-nanocomposites that can greatly lessen the impact of medical devices on the environment. In conclusion, bio-nanocomposites have great potential in the medical field, but they face several obstacles that must be solved before they can be widely implemented. They represent a new frontier in the study of medical materials due to their potential applications in areas as varied as drug delivery [109], tissue engineering [57], personalised medicine, enhanced diagnostics [202], and environmental sustainability.

## Conclusion

In conclusion, bionanocomposites are a well-established category of hybrid materials created by combining biopolymers, including chitosan, alginate, cellulose, starch, collagen, gelatin, pullulan, PHA, and PLA, with nanoscale fillers from various categories such as silicate-based materials (clays and silica nanoparticles), ceramics (nanohydroxyapatite), inorganic nanoparticles, synthetic layered double hydroxides, carbon-based nanomaterials (carbon nanotubes), and metal/metal oxides. This combination results in a bionanocomposite that exhibits high mechanical strength, biocompatibility, and bioactivity. Bionanocomposites with nanosized fillers are well-suited for drug delivery because they provide a complex diffusion route for encapsulated small molecules or drugs. This results in an effective barrier and prolonged release of the medication. Drug-releasing bio-nanocomposites are well-suited for wound dressing applications because they absorb water, lack toxicity, have strong adhesion to mucous membranes, and resilience to tearing. These properties make them an excellent choice for wound dressings. Bionanocomposites have emerged as a promising solution for various applications in emerging technologies such as matrix formation, drug delivery, tissue engineering, bone filler, dental applications, bioimaging, biosensors, and wound dressing. Despite the complexities associated with regulatory frameworks and manufacturing consistency, the future outlook for bio-nanocomposites remains optimistic, driven by their potential to enhance therapeutic outcomes, promote sustainable healthcare practices, and catalyse diagnostic and treatment modalities advancements. As we navigate towards a greener and more effective healthcare landscape, bio-nanocomposites stand as a beacon of innovation, offering a pathway towards transformative biomedical solutions that prioritise efficacy, sustainability, and patient-centric care.

## Data Availability

Not applicable.
